# Barriers to social participation for people with disabilities in NTD-endemic areas of Benin and Côte d’Ivoire: Assessing scope and associated factors

**DOI:** 10.1371/journal.pgph.0004104

**Published:** 2024-12-27

**Authors:** Yévèdo Borel Tossou, Paule Héléna Biaka, Fifamin Noël Christelle Gbaguidi, Vignon Bedie, Sonagnon Inès Elvire Agbo, Fernand Aimé Guedou, Kouakou Jerôme Kouadio, Sèdjro Gimatal Esaï Anagonou, Franck Zinsou Mignanwande, Parfait Djossou, Brunelle Vanessa Yemadje, Flora Sylvie Houndjrebo, Jeanne d’Arc Natacha Lessanh Arrawo, Yvette Faihoun, Harvey Johnson, Ghislain Emmanuel Sopoh, Jean Gabin Houezo, Mamadou Kaloga, Agui Sylvestre Dizoe, Mark Nichter, Anna Gine-March, Roch Christian Johnson

**Affiliations:** 1 Centre Interfacultaire de Formation et de Recherche en Environnement pour le développement Durable, Université d’Abomey-Calavi, Abomey-Calavi, Benin; 2 Institut des Sciences Anthropologiques de Développement, Université Félix Houphouet Boigny de Cocody, Abidjan, Côte d’Ivoire; 3 Institut Régional de Santé Publique de Ouidah, Université d’Abomey-Calavi, Abomey-Calavi, Benin; 4 Programme National de Lutte contre la Lèpre et l’Ulcère de Buruli, Benin; 5 Programme National de lutte contre l’Ulcère de Buruli, Côte d’Ivoire; 6 Programme National d’Elimination de la Lèpre, Côte d’Ivoire; 7 School of Anthropology University of Arizona, Tucson, Arizona, United States of America; 8 Anesvad Foundation, Bilbao, Spain; 9 Fondation Raoul Follereau, Paris, France; University of Ghana, GHANA

## Abstract

People with disabilities (PWDs) due to neglected tropical diseases (NTDs) or other causes experience restrictions on social participation (RSPs). This study aimed to investigate the magnitude of these restrictions and associated factors in NTD-endemic communities in Benin and Côte d’Ivoire. This cross-sectional quantitative and qualitative study was conducted from 2021 to 2022 among 841 people with disabilities (PWDs) and 90 community members and stakeholders. Questionnaires and interview guides were used for data collection. The World Health Organization P-scale score adapted to the local context, was used to assess RSPs. Univariate and multivariate analyses were performed to identify associated factors. Qualitative data were processed using triangulation or data comparison, categorized, cross-referenced, and synthesized by theme, hypothesis and indicator. Of the 841 PWDs, 65.9% had experienced RSPs. The median age (Q1; Q3) was 38 (22; 52) years, and the M/F ratio was 1.45. Of the respondents, 89.2% had a monthly income between 0 and 50,000 FCFA (76 euros); 43.7% were married, and 64.4% were uneducated. Only 98 (11.7%) were disabled due to NTDs. Factors (OR [95%IC], p-value) associated with RSPs were age (30 to 44 years (1.66 [1.06–2.59], p = 0.026), 45 to 59 years (2.26 [1.43–3.58], p = 0.001), and 60 to 74 years (2.35 [1.29–4.27], p = 0.005); Secondary/University level of education ((0.42 [0.28–0.65], p = 0.000); occupation (shopkeeper/housekeeper (0.40 [0.17–0.91]), p = 0.029), farmer (0.21 [0.11–0.40], p = 0.000), and other professions (0.44 [0.20–0.96], p = 0.038)); and income-generating activities (IGAs) (1.53 [1.06–2.22], p = 0.023). Our results demonstrate that the magnitude of RSPs among PWDs is high. The associated factors were age, education level, occupation, and IGAs.

## 1 Introduction

Disability is a global public health problem, a human rights issue and a development priority [1]. More than 1 billion people (15% of the world’s population) live with a disability, and nearly 200 million of these people have severe functional difficulties. The prevalence of disability is greater in developing countries [2]. Many obstacles prevent people with disabilities (PWDs) from accessing basic health and social services. Neglected tropical diseases (NTDs) are a major cause of disability in countries where they occur. NTDs encompass 20 conditions that affect 1.5 billion people worldwide, 40% of whom live in Africa [3]. Closely linked to poverty [4], lack of hygiene and sanitation, and difficult living conditions, NTDs lead to irreversible deformities in sufferers and are a source of disability [5]. For example, geo-helminthiasis and schistosomiasis can cause delayed physical and cognitive development in children. Leprosy, lymphatic filariasis, cutaneous leishmaniasis, and Buruli ulcer can lead to irreversible deformities, social exclusion, and stigmatization [6]. Onchocerciasis and trachoma, when inadequately managed, can lead to blindness [7].

In developing countries, there are major shortcomings in the management of disability caused by NTDs or other causes [8]. In many cases, health structures capable of providing effective care for people with physical impairments are lacking. Rehabilitation services ensuring the appropriate management of functional limitations are very limited [9]. In many cases, this results in restrictions on social participation (RSPs) for PWDs.

Indeed, social participation is based on values of acceptance and respect, on a sense of belonging and on the quality of the social links that people establish in normative social spaces: family, school, work, culture, leisure, community, and sociopolitical environments [10]. Social participation implies a reciprocal and egalitarian exchange between the individual and his or her community. It is based on a person’s fundamental need, expressed or otherwise, to be part of society and on the community’s willingness to welcome that person. It is built as much on the characteristics (emotional, cognitive, physical, spiritual, adaptive, etc.) of the participating person as on the resources and support measures provided by the community to its citizens [10]. It therefore follows that any social limitations or RSPs generate suffering, stigmatization. and even social exclusion.

The World Health Organization (WHO) has defined inclusive management of disability as a high-priority strategy in the fight against NTDs [8]. In Benin and Côte d’Ivoire, two West African countries where NTDs are endemic, the fight against these diseases is directed by dedicated national programs. According to national statistics for both countries, over 25% of new leprosy cases are detected with irreversible hand, eye, and foot disabilities [11, 12]. Over 30% of Buruli ulcer cases are detected with functional limitations of the limbs [13, 14]. In endemic communities, disability caused by NTDs often coexists with disability due to other causes. As a result, the management of disability in these two countries remains inadequate and fragmented. Only physical impairments are partially covered. Consideration of RSPs remains embryonic, if not nonexistent [15]. Moreover, it is ethically inadvisable for national programs to deal exclusively with disability due to NTDs. It is therefore essential to implement integrated and inclusive interventions.

The success of these interventions requires a thorough understanding of the essential characteristics of the disability, including the magnitude and associated factors [16, 17]. However, despite the importance of social participation in the lives of people with disabilities, whether due to NTDs or other causes, very few studies have been devoted to this subject.

The present study, to our knowledge the first of its kind, was conducted simultaneously in the two countries to investigate the magnitude of the restrictions on social participation among PWDs, whether due to NTDs or other causes, and associated factors in selected NTD-endemic communities in Benin and Côte d’Ivoire.

The results of this study provide guidelines for national programs in both countries to develop effective, holistic, context-specific, and evidence-based interventions to address RSPs experienced by PWDs.

## 2 Study settings and methods

### 2–1 Study settings

Data were collected in Benin in Ouidah, Lalo, Allada and Pobè and in Côte d’Ivoire in Divo, Yamoussoukro and Tiassalé. These localities were selected because they are endemic for NTDs, and most of them have specialized centers dedicated to treatment.

### 2–2 Study design

This cross-sectional study with mixed qualitative and quantitative methods occurred from September 2021 to September 2022 in Benin and Côte d’Ivoire.

A preparatory period preceded data collection in Benin and Côte d’Ivoire, during which the study protocol was submitted to ethics committees, data collection tools were developed and validated, and interviewers were recruited and trained. This period ran from September to November 2021 in Benin and from May to July 2022 in Côte d’Ivoire. Data collection was conducted from November 23^rd^, 2021 to January 24^th^, 2022 in Benin, and from July 31^st^ to August 09^th^, 2022 in Côte d’Ivoire.

### 2–3 Target populations

This study targeted a diverse range of participants, including individuals with disabilities—whether resulting from NTDs or other causes—categorized into four groups: motor, sensory, cerebral palsy, and multiple disabilities. Additionally, the study included community members, such as family members, opinion leaders, and caregivers.

#### 2-3-1 People with disabilities

The registers of the aforementioned care centers were examined to identify individuals with disabilities resulting from Neglected Tropical Diseases (NTDs) who had received treatment over the past five years. Their localities of origin were meticulously recorded. Subsequently, the research team visited the identified villages to locate and interview these individuals. A snowball sampling technique was employed to facilitate this process and made it possible to find both people with and without disabilities caused by NTDs.

#### 2-3-2 Community members and opinion leaders

Family members (ascendants or guardians) who provided consent were also included, as were people in charge of care at various levels (social workers, nurses, doctors and physiotherapists) as well as opinion leaders (village chiefs, religious authorities, and traditional healers).

#### 2-3-3 Sample size

Ultimately, the sample comprised of 841 PWDs, with 418 from Benin and 421 from Côte d’Ivoire, 90 community members participated, including 57 in Benin and 33 in Côte d’Ivoire.

### 2–4 Variables

In the context of this study, we defined ’RPS’ as a composite variable assessed based on the following elements:

- chances of finding work;
- frequentation of public places;
- participation in leisure and social activities (weddings, funerals, religious, participation in local or religious affairs);
- the possibility and feasibility of helping others (neighbors, friends, family members);
- the possibility of their opinion being considered during family discussions.

The explanatory variables are as follows:

- sociodemographic factors: age, sex, education, marital status and occupation;
- socioeconomic status: monthly income and income-generating activity (IGA);
- clinical: type of disability and illness.

### 2–5 Data collection tools

A questionnaire was drawn up, pretested and validated to collect quantitative data. The WHO P scale was used to support our investigation of RSPs. It was adapted to the local context and tested before the study. It comprises 18 items for which the interviewee is asked to indicate whether he or she perceives his or her level of participation as equal to that of his or her peers in each situation described.

For community members, an elaborate and pretested interview guide was used for in-depth interviews. A grid for observing interactions between PWDs and their community was also used to observe PWDs in their social context. To capture the experiences and interrelationships of PWDs with their families and communities, the interviewers spent 24 to 48 hours with the people selected for this in-depth study.

### 2–6 Data processing and analysis

Quantitative data from the 841 PWDs were collected using the KoboCollect digital tool, exported to Excel and checked for completeness and accuracy. Data analysis was performed using Statistical Package for Social Science (SPSS version 25). Quantitative variables were expressed as means with standard deviations, while qualitative variables were summarized as frequencies. Means were compared with Student’s t-test and frequencies with Pearson’s chi^2^ test or Fisher’s exact test, as appropriate. P-scale scores ranged from 0 to 90. Restriction levels were classified as follows: no restriction (0 to 12), mild restriction (13 to 22), moderate restriction (23 to 32), severe restriction (33 to 52) and extreme restriction (53 to 90) [18]. The variable was rendered binary, i.e., "No restriction" for the first two modalities (no restriction and mild restriction) and "With restriction" for the remaining three modalities (moderate restriction, severe restriction and extreme restriction). A bivariate analysis using logistic regression was performed, and variables with a p-value of less than 20% were retained for multivariate analysis. Multivariate analysis was performed using a stepwise iterative logistic regression model (Wald top-down). For all comparisons, the difference was considered significant for a p-value of less than 5%.

For qualitative data collection, interviews were conducted with the following target groups: healthcare staff, community members, and opinions leaders. These sessions were recorded, and the recordings were subsequently transcribed in French ensuring that the content was preserved in clear and accessible language. Transcribed data was coded to enable systematic categorization, cross-referencing, and triangulation. This approach facilitated thematic analysis guided by core study questions and hypotheses.

### 2–7 Ethical aspects

The study was approved by the Research Ethics Committee of the University of Parakou (CLERB-UP) in Benin under authorization N°0492/CLERB-UP/P/SP/R/SA dated 19/11/2021 and by the National Ethics Committee for Life Sciences and Health (CNESVS) of Côte d’Ivoire under authorization N/Réf: 076-22/MSHPCMU/CNESVS-kp dated 28/07/2022. The written informed consent was obtained from all participants included in this study. From the parent/guardian of each participant under 18 years of age, written informed consent was obtained.

## 3 Results

### 3–1 Participants

A survey was carried out among 841 PWDs, with 418 (49.7%) in Benin and 423 (50.3%) in Côte d’Ivoire. For the qualitative part of the survey, we interviewed

- 51 family members, with 35 in Benin and 16 in Côte d’Ivoire.
- 29 care staff, with 18 in Benin and 11 in Côte d’Ivoire; and
- 10 opinion leaders and stakeholders, with 4 in Benin and 6 in Côte d’Ivoire.

### 3–2 Characteristics of people with disabilities

The distributions of PWDs according to the different study variables in Benin and Côte d’Ivoire are shown in Table 1.

**Table 1:** Distribution of people with disabilities linked or not linked to NTDs according to different study variables in Benin and Côte d’Ivoire.

	Benin (%)	Côte-d’Ivoire (%)	Total (%)
**PEOPLE WITH DISABILITIES (n = 841)**			
**Restriction on Social Participation (RSPs)**	**n = 418**	**n = 423**	**n = 841**
Moderate to extreme	302 (72.2)	252 (59.6)	554 (65.9)
None or mild	116 (27.8)	171 (40.4)	287 (34.1)
**Age**	**n = 418**	**n = 423**	**n = 841**
[0–14]	63 (15.1)	45 (10.6)	108 (12.8)
[15–29]	112 (26.8)	81 (19.1)	193 (22.9)
[30–44]	89 (21.3)	120 (28.4)	209 (24.9)
[45–59]	96 (23.0)	105 (24.8)	201 (23.9)
[60–74]	47 (11.2)	46 (10.9)	93 (11.1)
75 and over	11 (02.6)	26 (06.1)	37 (04.4)
**Sex**	**n = 418**	**n = 423**	**n = 841**
Female	174 (41.6)	170 (40.2)	344 (40.9)
Male	244 (58.4)	253 (59.8)	497 (59.1)
**Schooling**	**n = 418**	**n = 423**	**n = 841**
Yes	94 (22.5)	201 (47.5)	295 (35.1)
No	324 (77.5)	222 (52.5)	546 (64.9)
**Education level**	**n = 411**	**n = 417**	**n = 828**
No level	317 (77.1)	216 (51.8)	533 (64.4)
Primary	51 (12.4)	107 (25.7)	158 (19.1)
Secondary	39 (09.5)	72 (17.2)	111 (13.4)
University	04 (01.0)	22 (05.3)	26 (03.1)
**Marital status**	**n = 355**	**n = 378**	**n = 733**
Single	119 (33.5)	142 (37.6)	261 (35.6)
Married/Couple	147 (41.4)	173 (45.8)	320 (43.7)
Divorced/Separated	46 (13.0)	20 (05.2)	66 (09.0)
widowed	43 (12.1)	43 (11.4)	86 (11.7)
**Profession**	**n = 355**	**n = 378**	**n = 733**
Civil servant	04 (01.1)	13 (03.4)	17 (02.3)
Craftsman/worker	49 (13.8)	28 (07.4)	77 (10.5)
Shopkeeper/Housekeeper	02 (00.6)	24 (06.3)	26 (03.5)
Farmers	04 (01.1)	43 (11.4)	47 (06.4)
Other	10 (02.8)	21 (05.6)	31 (04.2)
No profession	286 (80.6)	249 (659)	535 (73.1)
**IGA**	**n = 355**	**n = 378**	**n = 733**
Yes	106 (29.9)	109 (28.8)	215 (29.3)
No	249 (70.1)	269 (71.2)	518 (70.7)
**Monthly income** *	**n = 355**	**n = 378**	**n = 733**
[0–50000]	337 (94.9)	317 (83.9)	654 (89.2)
|50000–100000]	11 (03.1)	40 (10.6)	51 (07.0)
Over 100,000	07 (02.0)	21 (05.6)	28 (03.8)
**Type of disability**	**n = 418**	**n = 423**	**n = 841**
Motor disabilities	262 (62.7)	292 (69.1)	554 (65.8)
Sensory disability	95 (22.7)	78 (18.4)	173 (20.6)
Cerebral palsy	36 (08.6)	09 (02.1)	45 (05.4)
Multiple disabilities	25 (06.0)	44 (10.4)	69 (08.2)
**Causes**	**n = 418**	**n = 423**	**n = 841**
NTDs	49 (11.7)	49 (11.6)	98 (11.7)
Other illnesses	369 (88.3)	247 (58.4)	616 (73.2)
Don’t know	00 (00.0)	127 (30.0)	127 (15.1)

* The average income defined for the analyses is in the range 50000–100000. The minimum wage in Benin is 52,000 and 75,000 in Côte d’Ivoire. These values are found within |50000–100000]

The median age (Q1; Q3) was 38 (22; 52). In Benin, the median age (Q1; Q3) is 35 (20; 52) and in Côte d’Ivoire, it is 40 (26; 54). There was a significant difference between the two countries (p = 0.000). This difference was observed for both men (p = 0.005) and women (p = 0.043).

Among the PWDs, 497 (59.1%) were men and 344 (40.9%) were women (sex ratio = 1.45). In Benin, 244 (58.4%) were men and 174 (41.6%) were women (sex ratio = 1.40), whereas in Côte d’Ivoire, 253 (59.8%) were men and 170 (40.2%) were women (sex ratio = 1.48). There was no significant difference in sex distribution between the two countries (p = 0.672).

Analyses of school attendance were carried out only for PWDs aged 5 or older (school age). Of the 828 participants who met these criteria, 295 (35.6%) were enrolled in school: 158 (18.0%) in primary school, 111 (03.4%) in secondary school and 26 (03.1%) in higher education. The remaining 533 (64.4%) have never been to school [305 (62.1%) males and 228 (67.7%) females]. In Benin, 324 (77.5%) PWDs did not attend school. Of these, 193 (79.1%) were male and 131 (75.3%) females. In Côte d’Ivoire, 222 (52.5%) were not in school, of whom 118 (46.6%) were male and 104 (61.2%) females. PWDs were twice as likely to be enrolled in Côte d’Ivoire as in Benin, and the difference was significant (p = 0.000). This difference was equally significant for male (p = 0.000) and female (p = 0.005) school-age participants in both countries.

The analysis of marital status and occupation was limited to the 733 participants who were aged 15 and older. Among this subpopulation, 43.7% were married and 35.6% were single. In Benin, 147 (41.4%) of the participants were married, of whom 89 (44.1%) were men and 58 (37.9%) were women. In Côte d’Ivoire, 173 (45.8%) were married, of whom 125 (54.3%) were men and 48 (32.4%) were women. A significant difference was observed between the two countries (p = 0.003), and this difference was significant for both men (p = 0.013) and women (p = 0.000).

In terms of profession, 535 (73.1%) had no profession, and 198 (27.0%) declared that they had a profession. Among the latter, 77 (38.9%) were craftsmen/workers, 47 (23.7%) were farmers/peasants and 17 (08.6%) were civil servants. In Benin, 286 (80.6%) had no profession, of whom 149 (73.8%) were men and 137 (89.5%) were women. In Côte d’Ivoire, 249 (65.9%) had no occupation, of whom 123 (53.5%) were men and 126 (85.1%) were women. A significant difference was observed between the two countries (p = 0.000), and this difference was significant for both men (p = 0.000) and women (p = 0.003).

Among the PWDs, 215 (29.3%) had an IGA. In Benin, 106 (29.9%) had an IGA, of whom 41 (20.3%) were men and 65 (42.5%) were women. In Côte d’Ivoire, 109 (28.8%) had an IGA, of whom 62 (27.0%) were men and 47 (31.8%) were women. There was no significant difference between the two countries (p = 0.761).

In general, monthly income varied between 0 and 500,000 CFA francs (762 euros), with a median of 0 (0; 20,000). Over three-quarters; or 654 (89.2%) of the respondents, had a monthly income between 0 and 50,000 CFA francs (76 euros). In Benin, 337 (94.9%) had a monthly income of less than 50,000 CFA francs (76 euros). Of these, 189 (93.6%) were men and 148 (96.7%) were women. In Côte d’Ivoire, the proportion was 317 (83.9%), of whom 173 (75.2%) were men and 144 (97.3%) were women. The proportion of PWDs with a monthly income of less than 50,000 was significantly greater in Benin than in Côte d’Ivoire (p = 0.000). This difference was significant for men (p = 0.000) but not for women (p = 0.918).

In terms of the number of disabilities, 772 (91.8%) of the participants had a single disability, and the remaining 69 (08.2%) had multiple disabilities. A physical disability was found in 554 (65.8%) participants. Single sensory disabilities (visual, auditory, olfactory, or tactile) were found in 173 (20.6%) of the participants. Single cerebral palsy (CP) was found in 45 (05.4%) of the PWDs surveyed. In Benin, 393 (94.0%) of the PWDs surveyed had a single disability, of whom 230 (94.3%) were men and 163 (93.7%) were women, and 25 PWDs (06.0%) had multiple disabilities, of whom 14 (05.7%) were men and 11 (06.3%) were women. In Côte d’Ivoire, 379 (90.0%) had a single disability, of whom 229 (90.5%) were men and 150 (88.2%) were women, and 44 (10.4%) had multiple disabilities, of whom 24 (09.5%) were men and 20 (11.8%) were women. A significant difference was observed between the two countries (p = 0.000). This difference was significant for men (p = 0.000), but not for women (p = 0.116).

Only 98 (11.7%) of the PWDs surveyed had disabilities due to NTDs. In Benin, the number was 49 (11.7%), of whom 26 (10.7%) were men and 23 (13.2%) were women. In Côte d’Ivoire, the number was 49 (11.6%), of whom 30 (11.9%) were men and 19 (11.2%) were women. A significant difference was observed between the two countries (p = 0.000). This difference was significant for both men (p = 0.000) and women (p = 0.000).

### 3–3 Descriptive data of community members, opinion leaders and care staff

The distributions of community members according to the different study variables in Benin and Côte d’Ivoire are shown in Table 2.

**Table 2:** Distribution of family circles, resource persons, and care personnel according to sociodemographic variables in Benin and Côte d’Ivoire.

	Benin (%)	Côte-d’Ivoire (%)	Total (%)
FAMILY CIRCLE* **(n = 51)**			
**Age**	**n = 35**	**n = 16**	**n = 51**
[15–29]	01 (02.9)	02 (12.5)	03 (05.9)
[30–44]	12 (34.3)	03 (18.7)	15 (29.4)
[45–59]	17 (48.6)	06 (37.5)	23 (45.1)
[60–74]	05 (14.2)	05 (31.3)	10 (19.6)
75 and over	00 (00.0)	00 (00.0)	00 (00.0)
**Sex**	**n = 35**	**n = 16**	**n = 51**
Female	19 (54.3)	07 (43.7)	26 (51.0)
Male	16 (45.7)	09 (56.3)	25 (49.0)
**Schooling**	**n = 35**	**n = 16**	**n = 51**
Yes	18 (51.4)	07 (43.7)	25 (49.0)
No	17 (48.6)	09 (56.3)	26 (51.0)
**Education level**	**n = 35**	**n = 16**	**n = 51**
No level	17 (48.6)	09 (56.3)	26 (51.0)
Primary	08 (22.9)	04 (25.0)	12 (23.5)
Secondary	09 (25.6)	03 (18.7)	12 (23.5)
University	01 (02.9)	00 (00.0)	01 (02.0)
**Marital status**	**n = 35**	**n = 16**	**n = 51**
Single	02 (05.7)	03 (18.7)	05 (09.8)
Married/Couple	25 (71.4)	12 (75.0)	37 (72.5)
Divorced/Separated	03 (08.6)	00 (00.0)	03 (05.9)
Widowed	05 (14.3)	01 (06.3)	06 (11.8)
**Religion**	**n = 35**	**n = 16**	**n = 51**
No	02 (05.7)	04 (25.0)	06 (11.8)
Ancestral	11 (31.4)	03 (18.7)	14 (27.4)
Christian	22 (62.9)	08 (50.0)	30 (58.8)
Muslim	00 (0.00)	01 (06.3)	01 (02.0)
CARE STAFF** **(n = 29)**			
**Age**	**n = 18**	**n = 11**	**n = 29**
[15–29]	05 (27.8)	00 (00.0)	05 (17.2)
[30–44]	07 (38.9)	05 (45.4)	12 (41.4)
[45–59]	05 (27.8)	05 (45.4)	10 (34.5)
[60–74]	00 (00.0)	01 (09.1)	01 (03.4)
75 and over	01 (05.5)	00 (00.0)	01 (03.4)
**Sex**	**n = 18**	**n = 11**	**n = 29**
Female	10 (55.6)	05 (45.4)	15 (51.7)
Male	08 (44.4)	06 (54.5)	14 (48.3)
**Schooling**	**n = 18**	**n = 11**	**n = 29**
Yes	17 (94.5)	10 (90.9)	27 (93.1)
No	01 (05.5)	01 (09.1)	02 (06.9)
**Education level**	**n = 18**	**n = 11**	**n = 29**
No level	01 (05.5)	01 (09.1)	02 (06.9)
Primary	01 (05.5)	01 (09.1)	02 (06.9)
Secondary	02 (11.1)	01 (09.1)	03 (10.3)
University	14 (77.8)	08 (72.7)	22 (75.9)
**Marital status**	**n = 18**	**n = 11**	**n = 29**
Single	07 (38.9)	02 (18.2)	09 (31.0)
Married/Couple	11 (611)	08 (72.7)	19 (65.5)
Divorced/Separated	00 (00.0)	01 (09.1)	01 (03.4)
Widowed	00 (00.0)	00 (00.0)	00 (00.0)
**Profession**	**n = 18**	**n = 11**	**n = 29**
Doctor	03 (16.7)	00 (00.0)	03 (10.3)
Nurse/midwife	02 (11.1)	05 (45.4)	07 (24.1)
Physiotherapist	02 (11.1)	02 (18.2)	04 (13.8)
Social Assistant	04 (22.2)	01 (09.1)	05 (17.2)
Traditional healer	02 (11.1)	01 (09.1)	03 (10.3)
Other	05 (27.8)	02 (18.2)	07 (24.1)
RESSOURCE PERSON*** **(n = 10)**			
**Age**	**n = 04**	**n = 06**	**n = 10**
[15–29]	00 (00.0)	01 (16.7)	01 (10.0)
[30–44]	02 (50.0)	02 (33.3)	04 (40.0)
[45–59]	02 (50.0)	03 (50.0)	05 (50.0)
[60–74]	00 (00.0)	00 (00.0)	00 (00.0)
75 and over	00 (00.0)	00 (00.0)	00 (00.0)
**Sex**	**n = 04**	**n = 06**	**n = 10**
Female	00 (00.0)	01 (16.7)	01 (10.0)
Male	04 (100.0)	05 (83.3)	09 (90.0)
**Schooling**	**n = 04**	**n = 06**	**n = 10**
Yes	04 (100.0)	05 (83.3)	09 (90.0)
No	00 (00.0)	01 (16.7)	01 (10.0)
**Education level**	**n = 04**	**n = 06**	**n = 10**
No level	00 (00.0)	01 (16.7)	01 (10.0)
Primary	01 (25.0)	00 (00.0)	01 (10.0)
Secondary	02 (50.0)	02 (33.3)	04 (40.0)
University	01 (25.0)	03 (50.0)	04 (40.0)
**Marital status**	**n = 04**	**n = 06**	**n = 10**
Single	00 (00.0)	02 (33.3)	02 (20.0)
Married/Couple	04 (100.0)	04 (66.6)	08 (80.0)
Divorced/Separated	00 (00.0)	00 (00.0)	00 (00.0)
Widowed	00 (00.0)	00 (00.0)	00 (00.0)

* Family circle: These are relatives of PWDs: parents, other family members with whom they interact in one way or another on a basis daily (husband, wife, child, father, mother, brother, sister, cousin, etc.) or guardians.

** Care staff: These are social workers, disability educators, adaptive physical activity teachers, occupational therapists, nurses, physiotherapists, doctors (physical medicine and rehabilitation), psychologists, psychometricians, traditional healers and masseurs involved in the prevention or management of disability. These stakeholders provide formal assistance to PWDs, as opposed to family or extrafamilial contacts, who usually fall into the category of informal help.

*** Resource person: These are people with specific expertise in the field of disability or who are involved in defining strategies and interventions for PWDs (local elected representatives, etc.). They include academics specializing in different branches of the health social sciences who provide support to health professionals.

The majority, 38 (74.5%), of the interviewed family members were aged 30 to 59 years; 25 (49.0%) were men and 26 (51.0%) were women (sex ratio = 1). Regarding the level of education, 25 (49.0%) had attended school: 12 (23.5%) at the primary level, 12 (23.5%) at the secondary level and 1 (02.0%) at the tertiary level. The remaining 26 (51.0%) had no schooling. Regarding marital status, 37 (72.5%) were married. Regarding religion, 30 (58.8%) were Christians, and 14 (27.4%) practiced their ancestral religion.

The majority, 22 (75.9%), of the care staff were aged 30 to 59 years; 14 (48.3%) were men and 15 (51.7%) were women (sex ratio = 0.9). Regarding education level, 22 (93.1%) had received a university education. Regarding marital status, 19 (65.5%) were married. Of the care staff, 3 (10.3%) were doctors, 7 (24.1%) were nurses and midwives, 4 (13.8%) were physiotherapists, 5 (17.2%) were social workers and 3 (10.3%) were a traditional healer.

The majority, 9 (90.0%), of the community leaders were aged 30 to 59; 9 (90.0%) were men and 01 (10.0%) were women (sex ratio = 9). Regarding education level, 9 (90.0%) had attended school: 1 (10.0%) at the primary level, 4 (40.0%) at the secondary level and 4 (40.0%) at the tertiary level. Only 1 (10.0%) had not attended school. Regarding marital status, 8 (80.0%) were married.

### 3–4 Disability: Perspectives of care staff, community members and other stakeholders

#### 3-4-1 The supernatural origin of disability

Disability is attributed to multiple causes, ranging from the supernatural to the natural. Traditional healers and community members tended to interpret the birth of a disabled child as a response from the gods, viewing it as a punishment for the parents’ transgressions of cultural norms or violations of prohibitions. This perception is reflected in the words of Mr. YO.

*"There’s no such thing as chance in life, you know… . Everything that happens to you in life is something yourself have contributed to. If a family gives birth to a handicapped child, it’s because he or she has transgressed the rules and norms of the family, or even those of another family, and why not, of the divinities. Either one of the parents has made a promise to a deity which he or she has not honored, or it is the mother who, during her pregnancy, has not respected the prohibitions, or has stolen from a mined field, or has performed illegal abortions, etc. These are the children we call ’handicapped children.’ These children are often referred to as ’Tohossou.’ We used to have to return these children to their divinity. Today, however, things have changed, and they are taken care of and accepted by families…only their management is difficult. They are often a source of bad luck for their families"* (Mr. YO, 55, tradithérapeute, Lalo, Benin).

Other disabilities that develop over time, such as those caused by disabling illnesses like Buruli ulcer (BU), road accidents, and burns, may be perceived as having either supernatural origins, (bewitchment, spells, witchcraft) or natural causes (accidental falls, other disease). The perceived origin of the disability influences health care seeking and sacrifices deemed necessary to counter a spell or appease a spirit effecting not only the person with disability but their household.

#### 3-4-2 Rejection of people with disabilities

Supernatural perceptions of disability contribute to the rejection of persons with disabilities (PWDs) and exclusion from social life. In the communities of Benin and Côte d’Ivoire studied, those having disabilities are frequently rejected by community members and, in many cases, not regarded as complete human beings.

*"For the majority of the population, disabled people are nothing. People make fun of them and don’t consider them at all. In a family, when it comes to making decisions, for example, their voice is not taken into account because they are incapable and not autonomous. But others understand that it’s not their fault and sympathize with their pain. Disabled people have the same rights as everyone else, but because of their disability, they are neglected. They’re not considered the same as other normal people. Apart from that, people don’t give these people any special status, even though they have the same needs as normal people. As a result, they close in on themselves."* (Mr. TNI, 37, administrative, Divo, Côte d’Ivoire).

PWDs’ suffer from social rejection and discrimination which renders them socially and structurally vulnerable and a burden for their household as they are unable to contribute economically. As a consequence, they have little sense of agency, self-respect or social identity Mr. AI, Chef Quartier, described this situation:

*"* ***Disabled people are really pitiful. They are not autonomous; they are dependent on their parents, and their living conditions are miserable***. ***The person has the will to do something, but because of their disability, they cannot do anything. Economically speaking, disabled people*** *are not able to do anything, so they have to go into care. When we talk about care today, it is not an easy thing; it is about money, and they* ***do not produce anything to have resources.*** *Since* ***they are dependent on their parents****, financing them is complicated because the family does not have enough money either."* (Mr. AI, 51, Chef Quartier, Allada, Benin).

Perceived Role of the state in supporting persons with disability Persons with disabilities (PWDs) and their families face challenges that extend beyond biomedical care. Many respondents expressed the belief that the state should assume greater responsibility for supporting PWDs and helping them not only survive but thrive. For RD

*"The absence of regulatory legal instruments and adequate social assistance keeps these people and their families in poverty. The state must work to make the environment accessible to them and ensure that they are taken into account in training curricula. The government urgently needs to pay special attention to them. Apart from that, we need to take care of schoolchildren and pupils with disabilities, because when I was a teacher, there were cases like that, and because of the lack of maintenance and support, they dropped out of classes, even though very often they are very intelligent people"* (RD, 62, retired teacher, Pobè, Benin).

Healthcare staff working with persons with disabilities recognize that comprehensive psychosocial support is essential after hospital discharge. However, such care is currently unavailable, and the staffing and resources needed for it are spread thin across departments with limited coordination. According to Dame X, a social worker,

*"Nothing’s been done yet on the disability front. There is a lot of work to be done on disability. A disabled person you meet does not accept himself as a human being. To really take charge of them, we draw up a rehabilitation plan based on five components today: health, education, means of subsistence (income-generating resources), social, and finally the disabled person’s empowerment. It’s a cross-disciplinary approach. So today, when we take on a disabled person, we consider all these different aspects. That’s the only way we can try to improve their lives. There’s still a lot of work to be done. We’re clearing the way, but nothing’s done yet. Not only has nothing been done, but treatment is complicated! That’s because the specialists in charge are spread across several ministerial departments, such as the Ministries of Family, Health, and Professional Training. A situation that makes care even more complex. It is therefore necessary and imperative to centralize efforts to make care easier by creating a management unit"* (Dame X, 41, social worker, Ouidah, Benin).

Even with the implementation of a comprehensive home-based rehabilitation plan, there will still be cases where the social reintegration of cured patients remains a challenge, necessitating alternative government-supported care options.

*"As for the patients cured of leprosy, some are reintegrated into their families. Others are the home’s social cases. They don’t have anyone, because their families have rejected them, and they’re still the responsibility of our structure"* (SMA, 53, care center manager, Ouidah, Benin).

### 3–5 Restrictions on social participation

#### 3-5-1 Magnitude of restrictions on social participation

Among the PWDs surveyed, 47 (5.6%) had no RSPs, 240 (28.5%) had a mild restriction, 261 (31.0%) had a moderate restriction, 170 (20.2%) had a severe restriction and 123 (14.6%) had an extreme restriction. In Benin, 31 (07.4%) had no RSPs, 85 (20.3%) had a mild restriction, 153 (36.6%) had a moderate restriction, 97 (23.2%) had a severe restriction, and 52 (12.4%) had an extreme restriction. In Côte d’Ivoire, 16 (03.8%) had no RSPs, 155 (36.6%) had a mild restriction, 108 (25.5%) had a moderate restriction, 73 (17.3%) had a severe restriction and 71 (16.8%) had an extreme restriction.

Two-thirds, i.e., 554 (65.9%), of the PWDs surveyed suffered from an RSP. In Benin, 302 (72.2%) had such a restriction, compared with 252 (59.6%) in Côte d’Ivoire. The difference between the two countries was significant (p = 0.000). This difference was significant for men (p = 0.000) but not for women (p = 0.285).

RSPs can be observed in many areas, including access to employment, social isolation (in particular, whether an individual lives as part of a couple), access to leisure activities and the management of IGAs. All PWDs find it difficult to enjoy a fulfilling social life without discrimination. For the most part, they are subjected to mistreatment by their community and even some of their close relatives. As a result, PWDs are socially isolated, stigmatized, rejected, and lonely. This situation is illustrated by the words of Dame G, who, after becoming disabled due to Buruli ulcer (BU), faced rejection from both her community and close relatives.

*"I wasn’t born with a disability. It’s because of this disease called djomakou (BU) that I became like this! Without my two feet (motor disability) I’ve been seen as someone who can’t contribute anything to others. I’m ignored, and it’s as if I don’t exist. I’m now the victim of scornful looks and insults from others. This has ruined my chances of finding a loving husband. Even if they have the desire to get close to me, very quickly their friends, family and the gaze of others discourage them. For some men, it’s an experience to sleep with a disabled person. It’s humiliating and insulting. It’s bad… very bad…!"* (Dame G, 25, former disabled BU patient, Allada, Benin).

Mr. KWA from Côte d’Ivoire, disabled by polio, noted the extent to which individuals with disabilities are marginalized and treated as unwelcome in community activities.

*"We’re isolated by society. People don’t associate us with their program* […]. *It was a teacher’s wedding*. *We were invited*. *Some people prevented me from entering the hall. So, I withdrew*. *When I want to take part in activities, they refuse me*. *Others insult me with my evil*" (Mr. KWA, 28, disabled, Azopé, Côte d’Ivoire).

The type of one’s disability affects their ability to establish and maintain social relationships.

Responses from persons with different types of disabilities provided insights into challenges presented by different types of physical limitations. AF, a hearing-impaired individual, describes how he was separated from his partner due to both his disability and his inability to maintain affable social relationships within his household., *"I have difficulty communicating with those around me because of my disability. It wasn’t easy for me to find a woman. what can a person like me give to a woman? By God’s grace, I finally found a girl who liked me. She was really committed to staying with me, and I was really happy. Unfortunately, the people around me did everything they could to make her leave. Today, I’m alone … my wife has left me, not because of my handicap but because of other people" (Mr. AF, 32, hearing impaired, Divo, Côte d’Ivoire).*

Some persons with disabilities found social stigma and discrimination so difficult to bear that they preferred to be alone and self-isolated.

For example, X stated that "*I prefer to be alone to avoid being frustrated by the way others look at people with disabilities like me; I feel embarrassed in public*" (Dame X, 41, social worker, Ouidah, Benin).

The critical gaze of others affects not only persons with disabilities (PWDs), but also other members of their households.

The mother of a daughter suffering from CP confided,

*"You know!" (She shook her head, tried to hold back her tears and continued) I used to sell food at school. Since my daughter’s disability, everything has changed. Nothing was the same anymore. I don’t understand what happened. She was healthy at birth, could sit up on her own, and even crawl. Then, one day, I made my daughter sit up, and she couldn’t do it anymore. Ahan! What’s that? I do everything, but she just can’t do it. She falls down, and that’s it! […]… .. Because of her condition, I couldn’t sell food. No one wants to buy from the mother of an ’honnon’ (handicapped girl) … Sometimes I’m forced to entrust her to my mother to avoid taking her out in public … All sorts of things are said about me because of my daughter’s disability. Some think I offered my daughter as a sacrifice to a snake to prosper in my business and make lots of money. For others, her disability is a divine sanction"* (AH, 42, mother of a motor-impaired daughter, Ouidah, Benin).

#### 3-5-2 Factors associated with RSPs. *Bivariate analysis (Table 3)*

**Table 3:** Distribution of people with disabilities linked or not linked to NTDs according to the variables to be explained and RSPs in Benin and Côte d’Ivoire.

	Restrictions on social participation		OR**	95% CI**	p*
	Yes	No			
**Type of disability**	**n = 554**	**n = 287**	** **	** **	**0.028**
Motor	360 (65.0)	194 (35.0)	1	1	
Sensory	116 (67.1)	57 (32.9)	1.10	[0.76–1.57]	
CP**	38 (84.4)	07 (15.6)	2.92	[1.28–6.67]	
Multiple handicaps	40 (58.0)	29 (42.0)	0.74	[0.45–1.24]	
**Age**	**n = 554**	**n = 287**	** **	** **	**0.000**
[0–14]	53 (49.1)	55 (50.9)	1	1	
[15–29]	119 (61.7)	74 (38.3)	1.67	[1.04–2.69]	
[30–44]	139 (66.5)	70 (33.5)	2.06	[1.28–3.31]	
[45–59]	146 (72.6)	55 (27.4)	2.75	[1.69–4.49]	
[60–74]	72 (77.4)	21 (22.6)	3.56	[1.92–6.58]	
75 and over	25 (67.6)	12 (32.4)	2.16	[0.99–4.74]	
**Sex**	**n = 554**	**n = 287**	** **	** **	0,616
Male	324 (65.2)	173 (34.8)	1	1	
Female	230 (66.9)	114 (33.1)	1.08	[0.81–1.44]	
**Marital status**	**n = 501**	**n = 232**	** **	** **	0.241
Married/Couple	213 (66.6)	107 (33.4)	1	1	
Single	174 (66.7)	87 (33.3)	0.99	[0.70–1.41]	
Divorced/Separated	51 (77.3)	15 (22.7)	1.70	[0.90–3.19]	
Widow	63 (73.3)	23 (26.7)	1.37	[0.80–2.36]	
**Monthly income**	**n = 501**	**n = 232**	** **	** **	**0.010**
[0–50000]	457 (69.9)	197 (30.1)	1	1	
More than 50,000	44 (55.7)	35 (44.3)	0,54	[0,34–0,87]	
**Education level**	**n = 545**	**n = 283**	** **	** **	**0.000**
Out of school	376 (70.5)	157 (29.5)	1	1	
Primary	100 (63.3)	58 (36.7)	0.72	[0.50–1.05]	
Secondary/University	69 (50.4)	68 (49.6)	0.42	[0.29–0.62]	
**Profession**	**n = 501**	**n = 232**	** **	** **	**0.000**
No profession	384 (71.8)	151 (28.2)	1	1	
Civil servant	09 (52.9)	08 (47.1)	0.44	[0.17–1.17]	
Craftsman/Worker	60 (77.9)	17 (22.1)	1.39	[0.78–2.45]	
Shopkeeper/Housekeeper	13 (50.0)	13 (50.0)	0.39	[0.18–0.87]	
Cultivator	19 (40.4)	28 (59.6)	0.27	[0.14–0.49]	
Other	16 (51.6)	15 (48.4)	0.42	[0.20–0.87]	
IGA**	**n = 501**	**n = 232**	** **	** **	0.083
Yes	137 (63.7)	78 (36.3)	1	1	
No	364 (70.3)	154 (29.7)	1.35	[0.96–1.88]	

p*: p-value; CI**: Confident interval; OR**: Odds ratio; CP**: cerebral palsy; IGA**: Income-Generating Activity

The analysis reveals that several factors—such as type of disability, age, level of education, monthly income, occupation, and participation in income-generating activities (IGAs)—were associated with RSPs.

*Type of disability and RSPs*. A significant association was found between the type of disability and restrictions on participation in social life (p = 0.028). This association was observed for both men (p = 0.017) and women (p = 0.038). Additionally, cerebral palsy (CP) was identified as a risk factor for restricted social participation (OR = 2.92, 95% CI [1.28–6.67]).

In both Benin and Côte d’Ivoire, PWDs were identified who were unable to perform the most basic life tasks independently. A few examples may be cited to illustrate the sense of helplessness some of those experiencing disabilities feel.

Mr. AL, patient in Côte d’Ivoire confided,

*"I go a whole day without eating. I haven’t eaten anything since the morning*. *Because of my disability, I’m unable to eat by myself; I eat when my wife is available. She’s the person who also helps me bathe and dress myself* (Mr. AL, 25, disabled, Yacro, Côte d’Ivoire)

For Mr. KJ, handicapped by CP, his lack of autonomy and absence of a caretaker placed him in a precarious situation. He described having to go for days without washing or changing into clean clothes.

Similarly (KE) a woman disabled as a result of BU described how helpless she felt,

*"I can’t do my housework. I have one arm. I can’t do anything. It’s not pleasant, but for the moment, I can’t do anything. I try to do what I can, but I’m really limited. I wish I could take care of myself. It’s possible, isn’t it?"* (Ms. KE, 32, BU disabled, Tiassale, Côte d’Ivoire).

Mrs. AL, described how difficult cooking was for her and her embarrassment about needing help to go to the toilet:

*"Because of my disability, I can do almost nothing on my own. I find it difficult to cook; I cook when someone helps me. I’m also unable to have a bowel movement. I have a bowel movement in a pot, and someone else empties it. It’s a really difficult situation. Waiting for someone to help you empty the waste that’s come out of you… If we had toilets adapted to my situation, I think I’d already avoid making my family members feel uncomfortable, and I’d feel really good"* (Dame AL, 26, disabled, Tiassale, Côte d’Ivoire*)*

*Age as a factor associated with RSPs*. Overall, the risk of RSPs tended to increase with age (trend p = 0.000). This trend was consistent for both men (trend p = 0.000) and women (trend p = 0.033). Life stage presented different challenges. For example,

Mr. J, a person disabled with BU, described how he had to overcome his disability to feel like a man

*"It’s because of BU that I’m disabled in both legs. It is a situation I had a hard time coping with at the very beginning. Very few people accepted me. I did not have many friends. In addition, finding a wife was difficult. I was considered less than nothing. I am disabled! Yes! But I’m a man and I have desires and needs just like any other man. So, I have to take my life into my own hands and not let others totally destroy me because of their behavior!" (Mr. J, 45,* BU *disabled, Lalo, Benin).*

Mr. J was one of the few empowerment success stories that we encountered. He earned the esteem of community members by overcoming his limitations and becoming a productive member of society.

*Over time I’ve learned to live with my disability, and I’ve made my own way despite the rejections and insults of others. I’ve learned several trades. I’m a vulcanizer, a handyman-repairer, a motorcycle mechanic, a blacksmith, and a men’s hairdresser. I’ve got my own vulcanizer shop, which I run. My wife, who also helps me. And little by little, people began to accept me. I feel autonomous and loved by the members of my community. People even look up to me, even non-disabled people. I would have liked to expand my activities, but unfortunately, because of my disability, I don’t have access to microfinancing services. And because of my vulnerability, I’m often the victim of theft of work materials in my workshop. You see, it’s not nice. But they don’t have the power to discourage me as long as I’ve got my breath"* (Mr. J, 45, BU disabled, Lalo, Benin).

*Monthly income as a factor associated with RSPs*. The data revealed a significant inverse association between a monthly income above 50,000 FCFA (76 euros) and reported social perceptions (RSPs) (p = 0.010). This association was observed for men (p = 0.037) but not for women (p = 0.120). Thus, a higher monthly income appears to be protective against RSPs.

The qualitative interviews showed that most of the PWDs interviewed did not have sufficient means to meet their basic needs. For example, AY stated,

*"Being a disabled person like me without two feet isn’t easy. I have to eat, and I don’t want to be a beggar. That’s why, with the help of my family, I’ve been able to set up this little business. I sell a few things from home here. I’ve also learned to braid hair while watching others do it. But here again, it’s difficult; the customers have to stay on the floor so that I can braid their hair. Many don’t like it… . If I find funding or financial help, I can buy things and sell them to raise money to take care of myself and my child"* (Dame AY, disabled and reseller, Pobè, Benin*).*

*Level of education as a factor associated with RSPs*. There was a significant inverse association between education level and reported social participation (RSPs) (p = 0.000). This association was observed for both men (p = 0.001) and women (p = 0.012). The higher the level of education, the lower the probability of experiencing RSPs. Therefore, education serves as a protective factor against RSPs. ME’s aunt in Benin described how stopping school due to social stigma led to ME’s social isolation from peers.

*"My niece was going to school normally before her illness disabled her arm. After more than 3 years of treatment, what can she still do at school? For father, letting his daughter go to school is a waste of money. We’re currently looking for ways of putting her into an apprenticeship. And even then, what can she learn with one arm? Because of this situation, she no longer feels at ease with her old friends. no longer wants to open up to others because she feels diminished and fears the mockery of her peers and doesn’t want their pity"* (Dame ZY, 54, aunt and former BU patient, Allada, Benin).

We documented several cases like this while collecting illness narratives. We also encountered cases where higher levels of schooling served as a protective factor for PWDs. The case of G illustrates how education can serve as a protective factor:

*"G is an exceptional, dedicated girl. Despite her disability, she hasn’t let herself go. I got to know her two years ago when she was in CE2, and now she’s in CM2 preparing for her CEP exam. She’s a person with goodwill despite her disability, and does everything we ask her to do. Because we didn’t treat her differently because of her situation, she continues to be motivated. She’s determined to get her primary school diploma and continue until she has her BAC (first university diploma)"* (Sister AA, 39, nun, Pobè, Benin).

*Occupation and IGAs as factors associated with RSPs*. There was a significant association between occupation (p = 0.000) and RSPs. This association was observed for both men (p = 0.000) and women (p = 0.015).

Compared to nonprofessional PWD, those with a profession were at lower risk of developing RPSs except for those in the craftsman/worker occupational category.

In the multivariate analysis, in addition to occupation, income-generating activities (IGA) emerged as an associated factor (p = 0.023). Both IGA and various types of occupation serve as protective factors against RSPs, except for those in the artisan/worker category, which is a risk factor for RSPs. Most of the PWDs interviewed were unemployed, leading to a situation where they became a burden to their parents. The example of TA illustrates this point:

*Sometimes, when I find myself alone, I cry and ask God what sins I’ve committed, or what sins my parents have committed, to put them through all this misery. "I would like to have an income-generating activity that would enable me to take care of myself and provide for my own needs. I’d like institutions that take care of people with disabilities like me to come and get me and put me in homes for people with disabilities so that I can live with people like me because they’re the only ones who can understand what I’m going and that way, I won’t be a burden on my family* " (TA, 22, disabled and no longer at school, Pobè, Benin).

*Multivariate analysis*: *RSPs are a complex*, *multifactorial problem*. Multivariate analysis identified four factors associated with RSPs: age (p = 0.000), level of education (p = 0.000), occupation (p = 0.000), and IGA (p = 0.023) (Table 4).

**Table 4:** Distribution of people with disabilities booth linked or not linked to NTDs according to multivariate analysis on restriction of social participation in Benin and Côte d’Ivoire.

	Restrictions on social participation		ORa**	95% CI**	p*
	Yes	No			
**Age**	**n = 554**	**n = 287**	** **	** **	**0.004**
[15–29]	119 (61.7)	74 (38.3)	1	1	
[30–44]	139 (66.5)	70 (33.5)	1.66	[1.06–2.59]	**0.026**
[45–59]	146 (72.6)	55 (27.4)	2.26	[1.43–3.58]	**0.001**
[60–74]	72 (77.4)	21 (22.6)	2.35	[1.29–4.27]	**0.005**
75 and over	25 (67.6)	12 (32.4)	1.18	[0.53–2.59]	0.686
**Education level**	**n = 545**	**n = 283**	** **	** **	**0.000**
Out of school	376 (70.5)	157 (29.5)	1	1	
Primary	100 (63.3)	58 (36.7)	0.92	[0.59–1.44]	0.723
Secondary/University	69 (50.4)	68 (49.6)	0.42	[0.28–0.65]	**0.000**
**Profession**	**n = 501**	**n = 232**	** **	** **	**0.000**
No profession	384 (71.8)	151 (28.2)	1	1	
Civil servant	09 (52.9)	08 (47.1)	0.64	[0.22–1.83]	0.409
Craftsman/Worker	60 (77.9)	17 (22.1)	1.38	[0.77–2.49]	0.282
Shopkeeper/Housekeeper	13 (50.0)	13 (50.0)	0.40	[0.17–0.91]	**0.029**
Cultivator	19 (40.4)	28 (59.6)	0.21	[0.11–0.40]	**0.000**
Other	16 (516)	15 (48.4)	0.44	[0.20–0.96]	**0.038**
IGA**	**n = 501**	**n = 232**	** **	** **	**0.023**
Yes	137 (63.7)	78 (36.3)	1	1	
No	364 (70.3)	154 (29.7)	1.53	[1,6–2,22]	**0.023**

p*: p-value; CI**: Confident interval; ORa**: Adjusted Odds ratio; IGA**: Income-Generating Activity

RSPs increase with the age of the PWD (trend p = 0.000) (up to age 74). The level of education is inversely associated with RSPs, and the probability of experiencing RSPs decreases as the education level increases. Thus, subjects with primary education were 8% less likely than those with no schooling to suffer from RSPs (ORa: 0.92; 95% CI [0.59–1.44]; p = 0.723), and those with secondary education or higher were 58% less likely to suffer from RSPs (ORa: 0.42; 95% CI [0.28–0.65]; p = 0.000). Compared to having no profession, being in the trading and reselling profession reduced the probability of experiencing RPSs by 60% (ORa: 0.40; 95% CI [0.17–0.91]; p = 0.029), being in the farming profession by 79.0% (ORa: 0.21; 95% CI [0.11–0.40]; p = 0.000) and being in other professions (musician, volunteer, informal worker) by 56.0% (ORa: 0.44; 95% CI [0.20–0.96]; p = 0.038). Finally, the RPS score was 1.53 times higher in PWD who did not engage in an IGA than in those who did (ORa: 1.53; 95% CI [1.06–2.22]; p = 0.023).

Multiple factors interact leading to RSPs. Illness narratives collected during the study provided insights into how disabilities profoundly transform the lives of PWDs and their households throughout the life course. The case of Dame LO illustrates one such trajectory. (too long I have suggested cuts) *[Dame LO has a motor disability caused by*

*Buruli ulcer. She gets around on her buttocks because of her disability. Because of her illness, she was unable to finish school. When she was five years old and in the CP class, the disease put an end to her schooling. LO lost her father when she was hospitalized in Lalo. Raised by her mother, she was unable to afford an apprenticeship because of her situation. LO had few friends because of her disability. Many people did not want to be friends with her. Her disability made it difficult for her to find a partner. When a suitor came to her, community members made fun of him for being interested in a disabled woman. This eventually drove him away. At age twenty-five, which was already too late for most women in her community to marry, she met a man willing to marry her. When interviewed, she was carrying her first pregnancy. Her partner already had two wives, and she was the third wife. With no job to speak of, she lived off the income she earned from braiding hair, which she learned to do on the job… Much of the little income she earned was given to her mother who suffered from mental health problems. When interviewed, LO was interested in learning the hairdressing trade through some training course or apprenticeship. She saw this as a means to become independent and gain a sense of dignity and community (*Dame LO, 26 years old with a motor disability from BU, Lalo, Benin).

## 4 Discussion

The aim of this study was to assess restrictions on social participation (RSPs) for those persons with disabilities (PWDs) in rural communities in Benin and Côte d’Ivoire where skin neglected tropical diseases (NTDs) are endemic.

This study provided us with a significant of information on social restriction among PWD in Benin and Cote d’Ivoire. However, this study has some limitations. PWDs whose health had deteriorated may have already left their place of residence or traveled out of the community at the time of the survey. PWDs may also have overestimated their degree of social participation. Moreover, some subjects didn’t know the origin of their disabilities. This could be considered as a memory bias. Regarding the limitations during the interviews, some participants attitudes may have been exaggerated due to the presence of an observer, creating a bias.

The prevalence of RSPs documented in this study (65.9%) was significantly higher than rates reported in neighboring African countries, where surveys have focused on how PWDs experience individual NTDs. For example, a study by de Zeeuw *et al*. in Ghana found that 47.0% of former Buruli ulcer (BU) patients experienced RSPs [19]. This observation could be explained by the differences between the study populations. All types of disability person were integrated in this study, which is not the approach taken in de Zeeuw *et al* ’s study.

In the de Zeeuw *et al* study, patients in Ghana were reported challenges in meeting new people, giving their opinions in family discussions, being in long-term relationships, being socially active, and participating in recreational and social activities [19]. Monteiro *et al* reported that among cases of RSPs, mild restriction was more frequent (06.3%) [18] whereas Dahiru *et al* reported that most participants had RSPs, including severe (07.9%) and extreme restrictions (75.0%) [21]. These observations are similar to those made in Benin and Cote d’Ivoire, where PWD found it particularly difficult to be socially active, to give their opinions in family discussions, to travel outside the village, to take part in recreational and social activities, to help others, and to attend major festivals and rituals [19]. However, Beeres *et al* reported that former BU patients in Benin had more RSPs than former BU patients in Ghana. In both countries, most of the reported problems were related to sports, mainly playing with others, going to the playground and participating in sports at school [20]. The exploitation of the P scale in the work of de Zeeuw *et al* in 2014 also showed RSPs [21]. On the other hand, Vivian de Souza *et al* found that most patients in their 2016 WHO study had no PSR [22]. These observations can be explained by differences in context, such as the type of community support available in each country.

Age, education level, occupation, and IGAs were found to be associated with RSPs. In this study, the median age (Q1; Q3) of the PWD population was 38 (22; 52). The probability of experiencing RSPs increased with the age of the PWD (trend p = 0.000) in the univariate analysis. Multivariate analysis results show an increasing trend in the odds of RSP with age category up to 74 years. This suggests that while both young and old experience RSP, older PWDs are more affected by RSP compared to younger PWD. Nascimento *et al* in Brazil reported that PWDs had a mean age of 52 ± 1 years without a significant association with RSPs [20]. The difference observed between these studies may be linked to the nature of the target population in both studies, with a difference in size and age composition. The population in the Nascimento *et al* study was 263 versus 841 in the present study, and many other aspects such as differing social and cultural realities may explain these observations. In 2017, Reis *et al* in Brazil found a mean age of 51 ± 17 years [23]. In 2018, Rerreira *et al* in Brazil reported a positive and statistically significant association between social participation and the age of vulnerable people [24].

The study population was predominantly male (59.1%), with a sex ratio of 1.45 in favor of men. This observation is similar to Nascimento *et al* in Brazil, where Men were also predominant (50.2%) [25]. In 2017, Reis *et al* in Brazil found that the majority of participants were men (61.3%) and that sex was not associated with RSPs [23].

Among this subpopulation, married people predominated (43.7%), followed by single people (35.6%). Dahiru *et al* reported in 2022 that most (74.3%) participants were married [26]. In Abdela *et al* (2020), 69.2% of the participants were married. They also noted that single, divorced or widowed participants were associated with a 2.04 times greater risk of RSPs than married participants [27].

In the field of education, the present study shows that 64.9% of the surveyed PWDs were uneducated. Multivariate analysis results suggest that those with secondary education or university are less likely to experience RSP. In 2014, Nascimento *et al* in Brazil found that 43.0% of their participants had less than 8 years of education without a significant association with RSPs [25]. This could be explained by the fact that the educated person is more prone to social participation. Education can reduce social inequalities and promote social participation, because the educating person possesses a certain knowledge that is sometimes indispensable to the community. In 2017, Reis *et al* in Brazil found that education was not associated with RSPs [23]. And access to education was not always easy for the PWDs. In 2018, Rerreira *et al* in Brazil found a positive and statistically significant association between social participation and more years of formal education [24]. Ned *et al*. showed that the right to basic education was not respected for PWDs in Africa [15] and in 2017, Opoku *et al* found that PWDs in Cameroon faced serious obstacles to education [28]. We must therefore promote equitable access to education for PWDs.

The results of this study show that 73.1% of the PWDs surveyed had no profession, and 29.3% had an IGA. There was a significant association between occupation and experience of PWD (p = 0.000) in the univariate analysis and a statistically significant association with being a shopkeeper/dealer (p = 0.029), farmer (p = 0.000) and other occupations (p = 0.038). This finding aligns with those of Zeeuw *et al*., who reported that employment-related issues were the most frequently cited cause of RSPs and psychosocial distress among former Buruli ulcer (BU) patients in Benin and Ghana [19]. In their 2014 study in Brazil, Nascimento *et al* reported that 44.1% of PWD worked and found no significant association with RSPs [20]. De Souza *et al* found that patients in their study had limitations on daily activities [22]. Rerreira *et al* in 2018, reported a positive and statistically significant association between social participation and vulnerable people living with a partner in Brazil [24]. In 2017, Opoku *et al*. reported that PWDs in Cameroon faced serious barriers to employment [28].

In this study, the monthly income of the participants ranged from 0 to 500,000 FCFA (762 euros) and had a median of 0 (0; 20,000). Multivariate analysis results show that those with a monthly income more than 50,000 are less likely to experience RSP. In 2018, Rerreira *et al* in Brazil found a positive and statistically significant association between social participation and a higher socioeconomic position [24]. These observations clearly demonstrate the essential role played by professional integration and a monthly income possession in the well-being of PWDs.

In this research, 65.8% of the participants had a single physical disability, 20.6% had a single sensory disability, 05.4% had single CP and 08.2% had multiple disabilities. The high rate of physical disability may be linked to the preferential sites of NTDs. There was a significant association (p = 0.028) between RSPs and type of disability. PWDs with CP had experienced the most RSPs; the proportion of CP among PWDs who had experienced RSPs (04.3%) was approximately 3 times greater than that among PWDs who had not experienced such restrictions (01.4%). For other types of disability, the distribution of PWDs who had experienced RSPs was similar among those who had visible disabilities and those who did not. People suffering from CP have difficulty moving around, which may significantly restrict their social mobility and make them even more vulnerable to social isolation. Dahiru *et al* reported in 2022 that most of those surveyed had a disability (83.3%), and disability was more common among men than among women (95.0% vs. 86.1%) [26]. In 2014, Swartz reported that first-person accounts of disability and social exclusion were commonplace [29].

Our results clearly show that interventions relating to the management of disabilities through national programs should not be limited by medical aspects. These interventions must be as holistic as possible and take into account the factors identified in this study, namely, education, IGAs and community awareness, to create more opportunities for PWDs. Furthermore, in endemic communities, disabilities due to skin NTDs represent only 11% of disabilities. These results clearly show that the need for disability care extends well beyond skin NTDs and that this support must be as inclusive as possible. Perhaps some thought should be given to developing simple interventions that can benefit people with skin NTD-induced disabilities as well as disabilities with other causes.

## 5 Conclusion

This study documented the extent of restrictions on social participation (RSPs) among people with disabilities (PWDs) in rural communities of Benin and Côte d’Ivoire, where skin neglected tropical diseases (NTDs)are endemic and account for 11% of all disabilities. Community-based disability interventions must go beyond addressing immediate medical needs and address the psychosocial challenges faced by PWDs. Most of the PWDs surveyed experienced social exclusion, discrimination, loneliness, and stigmatization. Among the factors associated with RSPs, two stand out as particularly amenable to intervention: lack of education and limited access to income-generating activities (IGAs). National disability programs must invest in education and employment initiatives that provide PWDs with the resources not only to survive but also to thrive as productive socially accepted members of society.

## Supporting information

S1 Appendix Diagram of the final model showing the factors associated with PWDs in Benin and Côte d’Ivoire. (PDF)
