# Supplementary material for: Barriers to social participation for people with disabilities in NTD-endemic areas of Benin and Côte d’Ivoire: Assessing scope and associated factors
Source: PLOS Glob Public Health. 2024 Dec 27;4(12):e0004104. doi: 10.1371/journal.pgph.0004104 (PMC11676828; doi:10.1371/journal.pgph.0004104)
Supplement: S1 Appendix — (PDF) [file pgph.0004104.s001.pdf]

Final model

The results of this study show that age, education level, occupation and IGA are the factors influencing RSPs. National programs in both countries can develop effective, holistic interventions based on the final model.

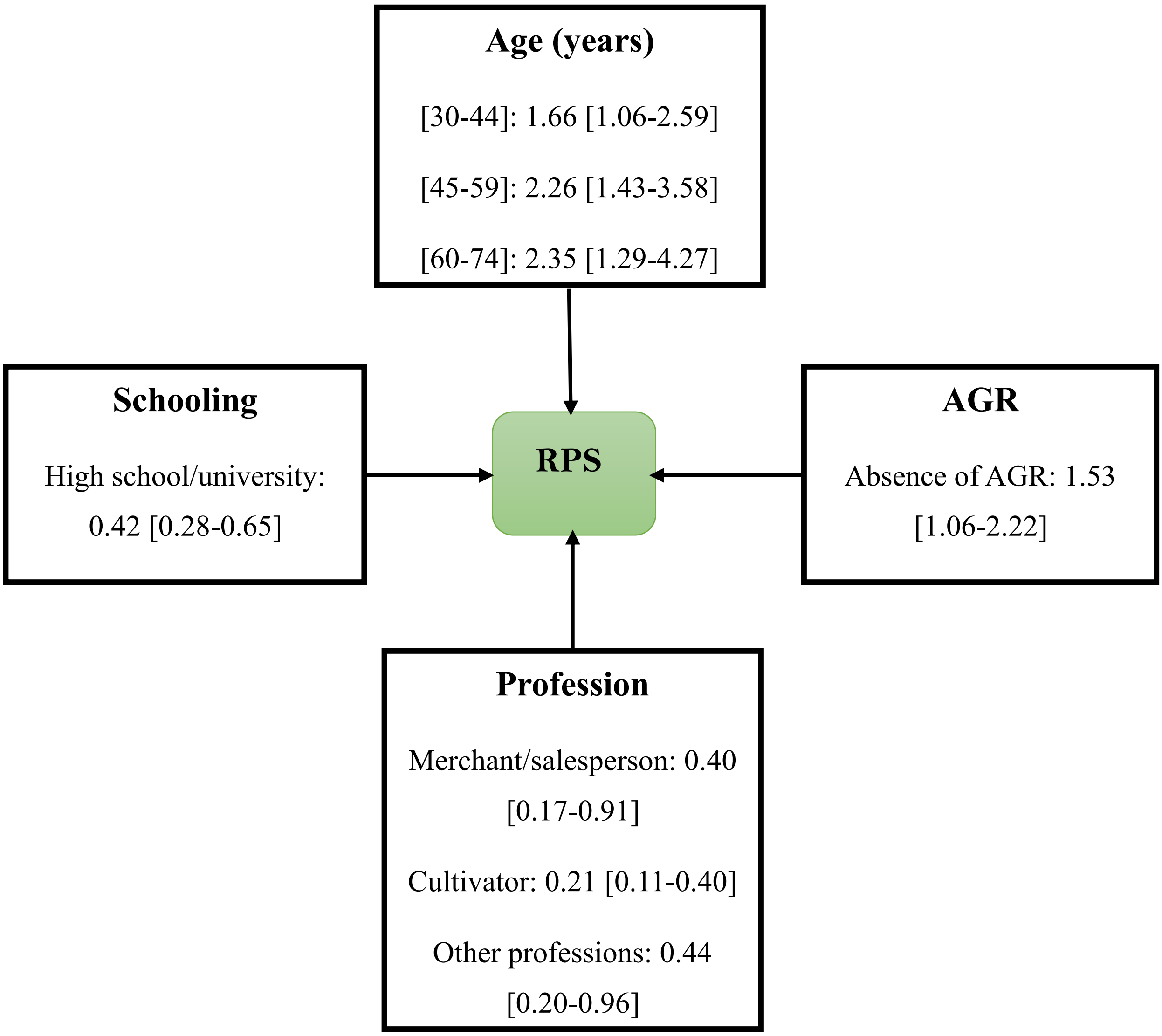

Figure 1: Diagram of the final model showing factors associated with RSPs for PWDs in Benin and Côte d'Ivoire.
